# Amyotrophic Lateral Sclerosis Prevalence Projection in 2040: A Less Rare Disease

**DOI:** 10.1002/acn3.70226

**Published:** 2025-10-14

**Authors:** Rosario Vasta, Stefano Callegaro, Antonio Canosa, Umberto Manera, Maurizio Grassano, Francesca Palumbo, Sara Cabras, Enrico Matteoni, Francesca Di Pede, Filippo De Mattei, Salvatore Tafaro, Neil M. Thakur, Ryan Grosenick, Fabiola De Marchi, Letizia Mazzini, Cristina Moglia, Andrea Calvo, Kuldip D. Dave, Adriano Chiò

**Affiliations:** ^1^ ALS Center, Department of Neuroscience “Rita Levi Montalcini” University of Turin Turin Italy; ^2^ Neurology 1, AOU Città della Salute e della Scienza di Torino Turin Italy; ^3^ Institute of Cognitive Science and Technologies National Research Council Rome Italy; ^4^ School of Advanced Studies, Center for Neuroscience University of Camerino Camerino Italy; ^5^ Thakur Health Sciences LLC USA; ^6^ The ALS Association Arlington Virginia USA; ^7^ ALS Center, Department of Neurology Azienda Ospedaliero Universitaria Maggiore della Carità, and University of Piemonte Orientale Novara Italy

**Keywords:** amyotrophic lateral sclerosis, epidemiology, prevalence, public health, survival

## Abstract

**Objective:**

To project ALS prevalence across multiple countries through 2040, accounting for both population aging and increased survival.

**Methods:**

Data from the Piemonte and Valle d'Aosta ALS register (PARALS) was used to estimate the trends in incidence and prevalence from 2005 to 2019. Survival trends over this period were also assessed. The observed annual increase was then projected into future years up to 2040. Concurrently, the incidence for each future year was calculated using population projections. Finally, the prevalence rate for each year was estimated as the product of the projected incidence and the projected survival. We also estimated survival for fifteen countries by dividing prevalence by incidence, based on available data, and applied the same increase observed in PARALS to project prevalence in these countries up to 2040.

**Results:**

Using data from 3294 patients, we determined that ALS survival increased by 0.06 years annually from 2005 to 2019 in Piemonte and Valle d'Aosta. Considering changes in incidence due to population aging, the prevalence is projected to reach 15.72 per 100,000 population by 2040 in this area, while rising by a median of 24.9% across multiple countries worldwide. If a new drug could provide a 6‐month increase in survival starting in 2025, disease prevalence would rise by 37.8% by 2040. We provided a web interface so users can model different data and assumptions.

**Interpretation:**

ALS prevalence is projected to increase significantly over the next decades. This underscores the need for careful planning and allocation of public health resources.

## 1 Introduction

While being the most common acquired motor neuron disease [1], Amyotrophic Lateral Sclerosis (ALS) absolute frequency is low. There is no single universal ALS frequency estimate, and its incidence and prevalence rates vary among countries, also based on the estimation method [2]. Registries provide continuous and comprehensive collection of ALS cases, being less susceptible to biases and offering the most reliable frequency estimates [3]. According to European ALS registries, ALS incidence ranges from 2 to 3/100,000 person‐years while its prevalence is 7–9/100,000 persons [3].

The temporal trend observed in incidence rates changes is likely attributable to an improved recognition of ALS cases, especially among older individuals, and to the inclusion of more subtle phenotypes, such as possible ALS and Frontotemporal Dementia‐ALS [3]. While the incidence rate itself may have remained relatively stable over recent decades, the number of incident cases is increasing due to the aging of the general population [4, 5].

Establishing the correct frequency of a particular disease and predicting potential changes is crucial for several reasons. In clinical practice, it impacts the differential diagnosis, making rare diseases less probable to be encountered. In public health, it guides the allocation of resources and helps understand the potential impact of one initiative over another.

While aging in the general population will drive the projected prevalence of ALS, the observed increase in survival [6]—if extended into the future—could further raise the number of ALS patients in the coming years. Further, regional prevalence will likely change at different rates as access to treatments and services varies across regions. If this is the case, then it would be prudent to be prepared for this scenario to ensure that the growing number of ALS patients receives appropriate care.

With this aim, in this study we analyzed the data from a long‐standing ALS register in order to estimate the expected ALS prevalence in 2040. We also developed an online tool that allows the user to calculate specific regional prevalence rates.

## 2 Methods

### 2.1 Prevalence Projection in the PARALS Area

Data from the Piemonte and Valle d'Aosta ALS Register (PARALS) were used to assess how prevalence has evolved. Using information from ALS Centers in the cities of Turin and Novara, as well as from other administrative sources, the PARALS collects data on ALS patients resident in the two Italian regions of Northern Italy since 1995 [5]. As such, it stands as one of the most enduring ALS Registers in Europe [3].

Relying solely on incident cases to estimate prevalence could result in underestimation, as it excludes patients from previous years who should contribute to the count of prevalent cases [7]. Based on our previous work, we know that a prevalence estimate calculated only from incident cases can be reliable when the study period exceeds 10 years, as it ensures the inclusion of nearly all long‐surviving patients [7]. Therefore, for the aim of this study, we considered patients diagnosed between 1995 and 2019 and estimated the prevalence only from 2005 onwards. Patients affected by Primary Lateral Sclerosis were excluded from the analysis.

Using the resident population of Piemonte and Valle d'Aosta, the annual crude point prevalence ratio was determined at December 31 of each year. The yearly incidence was also calculated. Following the formula linking prevalence to incidence (prevalence = survival × incidence) [8], the survival for each year was then computed by dividing the prevalence at the end of each year by the mean incidence over the period from 2005 to that specific year.

**FIGURE 1:**
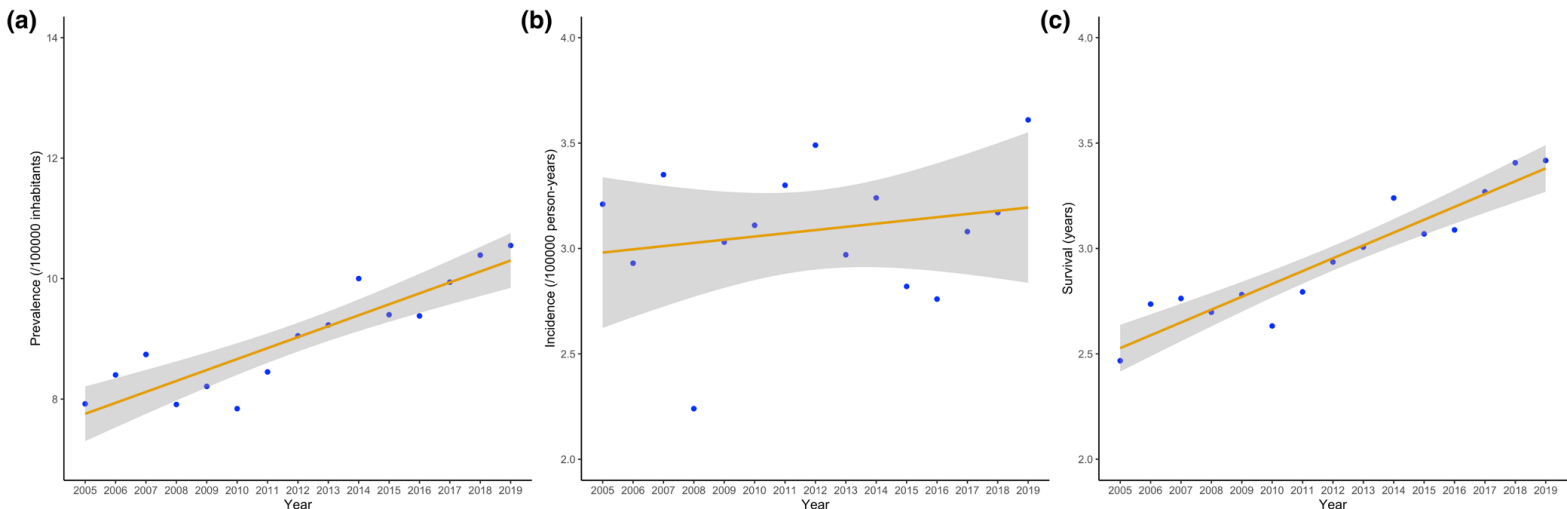
Trends in incidence (a) (*p*‐value = 0.461), prevalence (b) (*p*‐value = 0.000008), and survival (c) (*p*‐value = 0.0000002) for ALS in the Piemonte and Valle d'Aosta ALS Register (PARALS) from 2005 to 2019. Shaded areas refer to 95% CIs.

After confirming the assumptions for linearity, a linear regression model using the resulting survival estimates as the dependent variable and time as the independent variable confirmed that survival has increased linearly during the 2005–2019 period (Figure 1). The beta coefficient of time was utilized to estimate the annual increase in survival, while its *p*‐value was assessed for statistical significance (with a threshold of 0.05 indicating significance). Our recent findings indicate that survival rates have improved over time, with one plausible explanation being advancements in multidisciplinary care [6]. Assuming this hypothesis is correct, and that the beneficial impact of multidisciplinary care will continue to increase linearly over time, we applied the same growth rate from 2020 to 2040 to project future changes in survival.

Concurrently, due to the population aging, the proportion of people in the > 60 years age class is anticipated to increase more rapidly than other age groups. ALS mainly affects older individuals, with symptoms typically manifesting around the age of 65 [9]. Consequently, the number of new ALS cases is projected to rise faster than the general population, leading to an increase in ALS incidence [4]. Therefore, using population projections from 2020 to 2040 [10], we estimated the yearly incidence rate for this period. We assumed that the observed incidence rate within each age‐ (categorized as < 25, 25–35, 35–45, 55–65, 65–75, 75–85, and > 85 years) and sex‐specific subgroup would remain constant. In particular, the median incidence rate for each subgroup during the last five years of the study period (2015–2019) was applied to the projected population in each subgroup to estimate the expected number of cases for each year up to 2040. The overall incidence rate was then calculated by summing the expected cases across all subgroups, divided by the total population.

Finally, prevalence in each future year was computed as the product of the projected survival for that year and the mean of the incidence rates projected during the years leading up to it.

### 2.2 Prevalence Projection in Italy

We assumed that survival rates and sex‐ and age‐specific incidence rates in Italy in 2019 were identical to those observed in the PARALS dataset for the same year. The incidence rate was then projected by applying the median sex‐ and age‐specific incidence rates from the PARALS area during the 2015–2019 period to the projected Italian population [11]. This allowed us to estimate the total number of expected cases and, consequently, the projected incidence rate in Italy. The survival rate was assumed to increase at the same rate as in the PARALS area (0.06 years per year) from 2019 to 2040. Finally, the prevalence for each year from 2019 to 2040 was calculated as the product of the projected survival rate and the mean of the predicted incidence rates up to that year.

### 2.3 Prevalence Projection in Other Countries

Using population projections, we also projected the ALS prevalence in various countries by 2040. For Scotland and Northern Ireland, population projections were obtained from the Office of National Statistics website [12]. Projections for other countries were sourced from the International Database provided by the United States Census Bureau [13]. Prevalence and incidence estimates were retrieved from the literature [14, 15].

We calculated the resulting survival for each country by dividing the prevalence by the incidence, based on available estimates, and then applied the same increase observed in Piemonte and Valle d'Aosta for the years up to 2040. Due to the unavailability of the crude age‐specific rates, projecting incidence was not feasible; therefore, we assumed the last incidence rate available from the literature would remain constant over the time period. The prevalence was then calculated as the product of the projected survival and the incidence rate retrieved from the literature. Prevalence estimates were anticipated until both 2024 and 2040.

We selected studies that referred to national territories, opting for the most recent in cases of multiple studies. For China, we considered a study that covered multiple provinces, though not all, due to the large population it encompassed (430 million) [16]. We only considered countries for which prevalence and incidence estimates, as well as population projections, were available.

### 2.4 Edaravone

Edaravone has been approved for ALS patients in some countries. It has been suggested that treatment with edaravone could offer a 6‐month advantage at the two‐year mark [17]. However, the study recruited a small group of patients, and it is uncertain if the beneficial effect will persist beyond this timeframe [18]. Despite a recent phase III randomized clinical trial of an oral edaravone formulation not meeting primary or key secondary endpoints, we chose to incorporate the potential effect of edaravone on survival into our projections for the USA and Japan. This addition corresponded to an extra 0.5 years from the marketing year, which was 2017 in the USA and 2015 in Japan.

### 2.5 Ethics Approval

The study was approved by the Ethical Committee of the Turin ALS Center (Comitato Etico Azienda Ospedaliero‐Universitaria Città della Salute e della Scienza, Torino, #0038876).

## 3 Results

A total of 3294 ALS patients were diagnosed from 1995 to 2019 in Piemonte and Valle d'Aosta. The demographical and clinical characteristics were similar to previous findings from other registry‐based studies and are reported in Table 1.

**TABLE 1:** Demographical and clinical characteristics of the ALS cohort from the PARALS.

	PARALS cohort (*n* = 3294)
Sex, M (%)	1809 (54.9)
Onset age, years (median, IQR)	67.74 (60.07–73.92)
Onset site (%)	
Bulbar	1153 (35.0)
Cognitive	3 (0.1)
Respiratory	60 (1.8)
Spinal	2077 (63.1)
Diagnostic delay, months (median, IQR)	9.57 (5.57–13.67)
Survival, months (median, IQR)	20.00 (9.47–37.50)
Patients alive at the end of the study period (%)	213 (6.5)

*Note:* Cognitive onset refers to those patients who presented with cognitive impairment as their first symptom, ranging from dysexecutive impairment to a frontotemporal dementia. Survival for calculated for the subset of patients who deceased during the study as the time from diagnosis to death.

The number of prevalent cases increased from 350 at the start to 468 by the end of the study period (Table S1). Accordingly, the crude prevalence ratio exhibited a steady rise, starting at 7.92 per 100,000 population on December 31, 2005, and reaching 10.55 per 100,000 population on December 31, 2019, with an annual increment of 0.182 points (*p* = 0.000008). Incidence ranged from 3.21 in 2005 to 3.61 in 2019, while not exhibiting a linear trend in between (*p* = 0.461). Finally, the median survival during the same period increased from 2.47 to 3.42 years, showing an annual increment of 0.06 years (*p* = 0.0000002) (Figure 1), in line with our previous study [6].

By applying the same increase for each year up to 2040, the median survival is anticipated to reach 3.72 years in 2024 and 4.67 years in 2040. Concurrently, due to the aging population in Piemonte and Valle d'Aosta, the incidence rate is expected to rise from 3.61 per 100,000 person‐years in 2019 to 3.36 in 2024 and 3.82 by 2040. Consequently, the prevalence of ALS in these regions is anticipated to increase, reaching 11.70/100,000 population in 2024 and 15.72 by 2040, corresponding to an estimated 654.5 ALS patients alive as of December 31, 2024 (Table S1).

The Italian population is projected to globally decrease from the observed 59,816,673 residents on January 1, 2019, to an anticipated 56,329,104 by January 1, 2040. This decline is attributed to a proportionally greater decrease in younger age groups compared to the increase in older age groups (Table S2 and Figure S1). Consequently, the ALS incidence rate is expected to still rise across the national territory, from 3.01/100,000 person‐years in 2019 to 3.19 in 2024 and to 3.78 by 2040. By applying the same increase rate of survival observed in the PARALS register, the prevalence rate is forecasted to increase from 10.28/100,000 population (6147.5 prevalent cases) in 2019 to 11.54 (6808.2 prevalent cases) in 2024 and 15.91 (8963.9 prevalent cases) by the end of 2040 (Table S3).

In the United States of America, prevalence is expected to rise from 9.10/100,000 population in 2018 to 9.68 in 2024, reaching 11.21 in 2040. This increase will result in 8778 and 12,137 more patients nationwide with respect to 2024 and the last estimate provided, respectively. Considering the potential beneficial effect of edaravone, the prevalence could further increase to 12.01/100,000 population.

**FIGURE 2:**
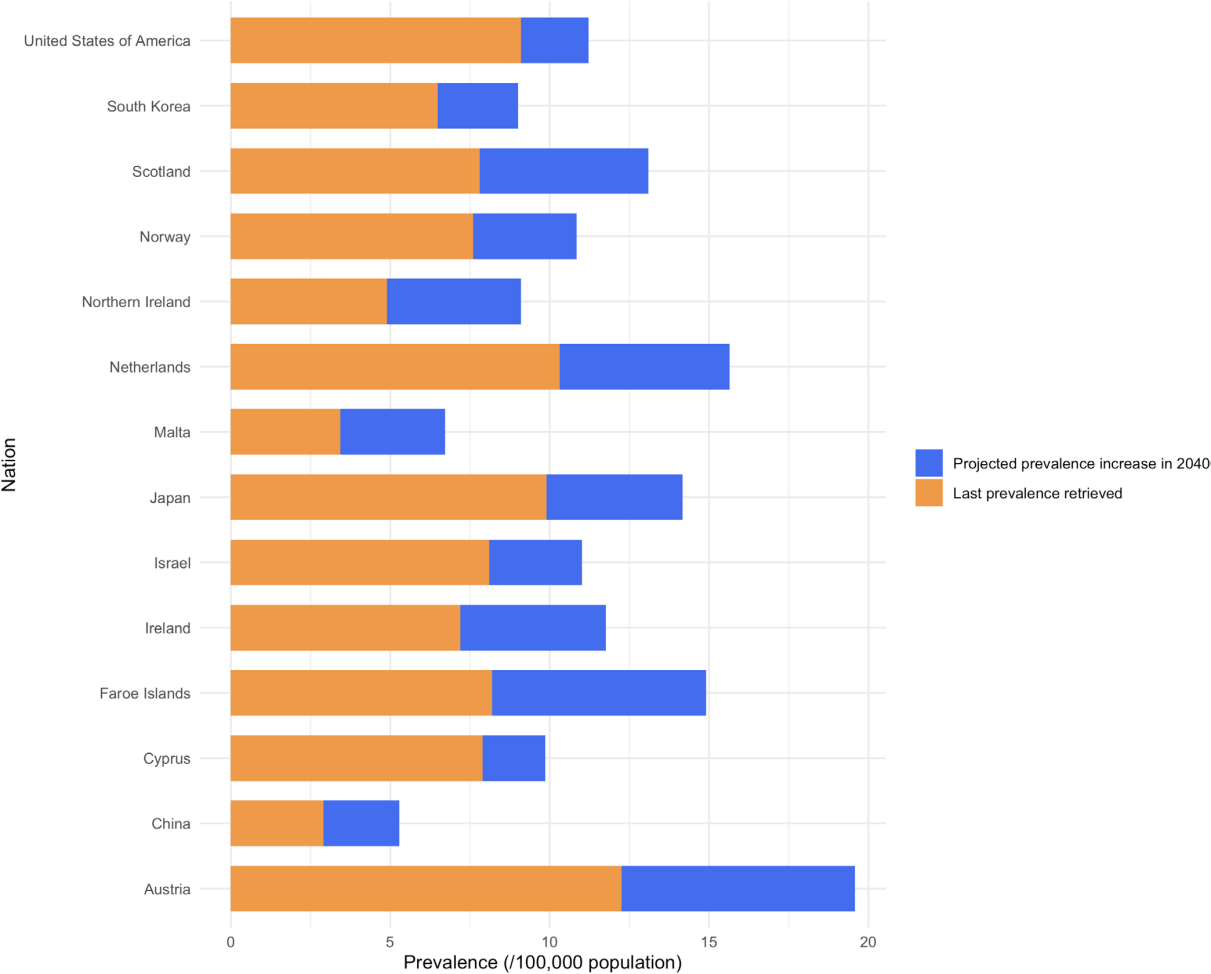
Projected ALS prevalence for multiple countries from the most recent estimates available in the literature to 2040.

Similarly, in Japan, the point prevalence in 2009 was 9.90/100,000 population. This was anticipated to have increased to 11.96 in 2024 and is projected to reach 14.18 by 2040, resulting in 16,033.4 prevalent cases (an increase of 3386.1 and 1298.5 cases compared to 2009 and 2024, respectively) (Table 2 and Figure 2). Factoring in the effect of edaravone, the prevalence in Japan could further rise to 15.32/100,000 population.

**TABLE 2:** Prevalence projections for several countries world widely.

Nation	Estimates from literature						Projections in 2024				Projections in 2040				
	Year	Population	Prevalence	Prevalent cases	Incidence	Survival	Population	Survival	Prevalence	Prevalent cases	Population	Survival	Prevalence	Prevalent cases	Prevalence increase (%)
Austria [19]	2011	8,475,092	12.26	1039.0	4.20	2.92	8,967,982	3.70	15.54	1393.6	9,275,403	4.66	19.57	1815.0	25.9
China [16]	2016	1,382,148,409	2.91	40,220.5	1.65	1.76	1,416,043,270	2.24	3.70	52,393.6	1,407,276,464	3.20	5.28	74,388.6	42.7
Cyprus [20]	2014	1,172,653	7.90	92.6	1.26	6.27	1,320,525	6.87	8.66	114.4	1,431,463	7.83	9.87	141.2	13.9
Faroe Islands [21, 22]	2009	48,852	8.20	4.0	3.60	2.28	52,933	3.18	11.45	6.1	56,866	4.14	14.90	8.5	30.1
Ireland [23, 24]	2013	4,677,112	7.20	336.8	2.82	2.55	5,233,461	3.21	9.05	473.6	5,848,514	4.17	11.77	688.3	30.1
Israel [25]	2013	7,803,834	8.10	632.1	1.80	4.50	9,402,617	5.16	9.29	873.5	11,850,653	6.12	11.02	1305.5	18.6
Japan [26]	2009	127,750,918	9.90	12,647.3	2.30	4.30	123,201,945	5.20	11.96	14,734.9	113,086,546	6.16	14.18	16,033.4	18.6
Malta [27]	2018	449,044	3.44	15.4	2.48	1.39	469,730	1.75	4.34	20.4	486,347	2.71	6.71	32.7	54.6
Netherlands [28]	2008	16,410,055	10.32	1693.5	2.77	3.73	17,772,378	4.69	12.99	2308.6	18,476,330	5.65	15.64	2889.4	20.4
Northern Ireland [29]	2005	1,727,700	4.90	84.7	2.00	2.45	1,914,000	3.59	7.18	137.4	1,912,000	4.55	9.10	174.0	26.7
Norway [30, 31]	2015	5,192,607	7.60	394.6	2.17	3.50	5,509,733	4.04	8.77	483.2	5,980,508	5.00	10.86	649.2	23.8
Scotland [32]	2017	5,420,000	7.81	423.3	3.83	2.04	5,496,877	2.46	9.42	517.8	5,513,969	3.42	13.1	722.3	39.1
South Korea [33, 34]	2015	50,646,419	6.49	3287.0	1.68	3.86	52,081,799	4.40	7.39	3848.8	52,189,080	5.36	9.01	4702.2	21.9
USA [15, 35]	2018	326,838,199	9.10	29,742.3	1.60	5.69	341,963,408	6.05	9.68	33,102.1	373,527,973	7.01	11.21	41,880.0	15.8

*Note:* Prevalence increases refer to the comparison of 2040 estimates with 2024 estimates.

Prevalence rate is expected to increase across all other countries considered, with a median percentage rise of 24.9% from 2024 to 2040 and of 55.6% from last retrieved estimates to 2040. However, the impact will vary based on population size. China is expected to exhibit one of the highest increases in prevalence (42.7%). Given its population size, this increase translates to 21,995 more patients from 2024 to 2040. Conversely, the 54.6% increase that is projected to be seen in Malta will translate into 12.3 more patients from 2024 to 2040 (Table 2 and Figure 2).

## 4 Discussion

Using data from a long‐standing register, we confirmed that ALS survival has improved, and accordingly its prevalence has increased from 7.92 to 10.55 per 100,000 population between 2005 and 2019. This study was not designed to infer causality, so we cannot definitively determine the reasons for the observed increase in survival. However, we believe that the growing number of patients followed by tertiary centers, as well as the enhancement in the multidisciplinary care provided within these centers, could be among the plausible explanations [6].

Assuming this increasing trend in survival will continue into the future, we estimated the ALS prevalence to further increase, reaching 15.72 per 100,000 population in Piemonte and Valle d'Aosta by 2040. Across multiple countries, these projections indicate an expected average increase of 24.9% from 2024 to 55.6% compared to the most recent available estimates.

ALS prevalence has previously been projected to rise significantly. By projecting the age‐ and sex‐specific incidence and survival rates to future populations, researchers have concluded that the number of ALS patients will increase by 20% in Europe and by 34% in the USA from 2015 to 2040, and globally by 31% in the ten countries primarily considered in the study [4]. Also, a recent study projected that, based on the aging population, the number of ALS cases in the USA will increase by 10% between 2022 and 2030 [36].

These projections are based on the anticipated increase in the number of at‐risk individuals due to the aging population. However, as discussed in these studies [4, 37], these estimates are likely conservative as patient survival has improved over time. To our knowledge, our study represents the first comprehensive projection of ALS prevalence over the next 15 years incorporating changes in both incidence and patient survival. Accordingly, our analysis projects a similar 30% increase in ALS prevalence to that reported in a previous study across multiple countries [4], but over the shorter period from 2024 to 2040. This indicates a greater absolute increase in our projections, as the starting point is shifted ten years later.

Unlike cardiovascular diseases, our limited understanding of the multifactorial nature of ALS etiology means these projections will not help prevent the increase in the number of cases. Thus, while currently of no use in guiding prevention efforts, prevalence projections could be crucial for the proper and timely allocation of public health resources.

To address the growing number of patients, more ALS tertiary centers should be established and equipped across national territories. In fact, this is already an ongoing process, and some regions are making particular efforts to expand ALS tertiary centers and their utilization [38]. This expansion will, in turn, likely contribute to improving patient survival, further increasing the absolute number of individuals living with ALS at any given time. The timing and scale of these efforts should correspond to the additional number of cases expected based on the country's size. For example, in the USA, the annual cost per ALS patient is approximately $70,000 [39]. Factoring in this figure for thousands of patients translates to a significant rise in overall expenses, necessitating a timely allocation of resources. Additionally, within each tertiary center, internal organization should be adapted to accommodate the anticipated increase in the number of patients requiring care.

In order to adapt to differences across countries and regions, we made our model freely accessible online for researchers and physicians (https://preals.als.org/). Users have the flexibility to input their own data for projections, while default values based on ALS survival and incidence estimates from the PARALS register are also provided. The creation of the website will allow users to make projections for countries originally not included in the study and for scenarios of their own devising. For example, the website provides the option to incorporate tertiary center use into the final survival estimates for a specific region.

Certainly, we had to apply certain constraints to proceed with the analysis, and two main assumptions pose significant limitations to this study. First, we assumed that survival would continue to increase at the same rate until 2040, potentially due to the beneficial effects of multidisciplinary care, among other factors. However, it could be argued that such improvement could plateau after a certain number of years. Second, we assumed such an increase would be applicable to the entire national territory rather than just a restricted area served by a tertiary ALS center. This assumption was dictated by the fact that nearly all individuals in the PARALS cohort received care at ALS specialty clinics. As a result, we were unable to disentangle the survival benefit specifically associated with clinic attendance. Additionally, data on the proportion of patients followed by tertiary centers at a national level are not available for all the countries included in this study. Although these assumptions could theoretically have led to an overestimation of the projected prevalence, there are multiple reasons why these findings might actually have resulted in an underestimation. Firstly, estimates from multiple countries with shorter study periods than PARALS and that were not based on registries are likely underestimated [7]. For example, the USA reported a significantly lower incidence rate (1.6/100,000 person‐years) compared to the average of ALS registries. Assuming this discrepancy is due to incomplete case ascertainment and applying the most recent PARALS incidence rate (3.09/100,000 person‐years), the anticipated prevalence would reach 13.18 by 2040, representing a 44.8% increase compared to 2018. Furthermore, we excluded patients affected by Primary Lateral Sclerosis. However, these patients will contribute to the burden on ALS tertiary centers in the coming years. At the time of writing this paper, there is also no evidence on the effect of tofersen on the survival of patients with SOD1‐ALS [40, 41]. Therefore, this effect was not considered in our analysis. However, given that only a small proportion of ALS patients (approximately 2%–3%) carry a pathogenetic mutation in the *SOD1* gene, this underestimation is expected to have a negligible impact on the overall projection. Finally, new drugs could become available between now and 2040. For example, if a new drug able to extend ALS survival by an average of 6 months becomes available between now and 2040, the prevalence estimates across the countries considered in this study would increase by a median of 37.8% from 2024 to 2040. If available, users will have the option to input data about tofersen or other drugs on the website.

We also assumed that the incidence rate across each age and sex‐specific subgroup will remain stable through 2040. In fact, we believe that over such a short period, the distribution of causal factors is unlikely to change significantly. There are prevention studies in progress [42], and even case reports suggesting genetic forms of ALS have been prevented [43], but these trials are predominantly focused on rare genetic forms of ALS and are unlikely to impact the overall prevalence. Furthermore, our current knowledge of environmental risk factors does not yet provide actionable targets to reduce the disease's incidence [9].

We were also unable to project the prevalence in other countries, due to the lack of nationwide ALS prevalence and incidence estimates. Previous research indicates that some of these regions, such as India and the African continent, will experience the most rapid aging [4]. While we can hypothesize that survival will not increase uniformly within these countries, the higher incidence rate alone will contribute to a higher number of individuals with ALS. Therefore, worldwide, the projected ALS prevalence is likely to increase even more rapidly than what has been depicted in this study. However, the website tool will allow users to adjust projections based on each country's specific variables.

Lastly, it should also be noted that the further increase in survival we calculated based on the putative effect of edaravone in countries where it is available should be interpreted with caution, given the negative results from previous observational and experimental studies.

In conclusion, ALS prevalence is projected to substantially increase over the next 15 years. Public health policies should promptly allocate resources to ensure these patients receive the necessary care until a cure is found. We provide a framework to help model those increases and the impacts of changes in survival.

## Author Contributions

Rosario Vasta contributed to the conception and design of the study, to the acquisition and analysis of data, and to the drafting of the text and preparing the figures. Kuldip D. Dave, Neil M. Thakur, and Adriano Chiò contributed to the conception and design of the study. Stefano Callegaro contributed to the design of the study and to the website development. Ryan Grosenick contributed to the website development and to the drafting of the text. Antonio Canosa, Umberto Manera, Maurizio Grassano, Francesca Palumbo, Sara Cabras, Enrico Matteoni, Francesca Di Pede, Filippo De Mattei, Salvatore Tafaro, Fabiola De Marchi, Letizia Mazzini, Cristina Moglia, Andrea Calvo, and Adriano Chiò contributed to the acquisition of data and to drafting of the text.

## Conflicts of Interest

The authors declare no conflicts of interest.

## Supporting information


**Figure S1:** Projected Italian population trends from 2019 to 2040, stratified by age group.
**Table S1:** Incidence, survival and prevalence projections over the PARALS area.
**Table S2:** Distribution of Italian population by age and sex, as it was observed in 2019 and anticipated in 2040.
**Table S3:**Incidence, survival and prevalence projections over the entire Italian national territory.

## Data Availability

Data and code for statistical analysis are available upon reasonable request by interested researchers.
