# Supplementary material for: Amyotrophic Lateral Sclerosis Prevalence Projection in 2040: A Less Rare Disease
Source: Ann Clin Transl Neurol. 2025 Oct 14;13(2):379–86. doi: 10.1002/acn3.70226 (PMC12883675; doi:10.1002/acn3.70226)
Supplement: Supplementary file 1 — Figure S1: Projected Italian population trends from 2019 to 2040, stratified by age group. Table S1: Incidence, survival and prevalence projections over the PARALS area. Table S2: Distribution of Italian population by age and sex, as it was observed in 2019 and anticipated in 2040. Table S3:Incidence, survival and prevalence projections over the entire Italian national territory. [file ACN3-13-379-s001.docx]

**Supplementary figure 1.** Projected Italian population trends from 2019 to 2040, stratified by age group.


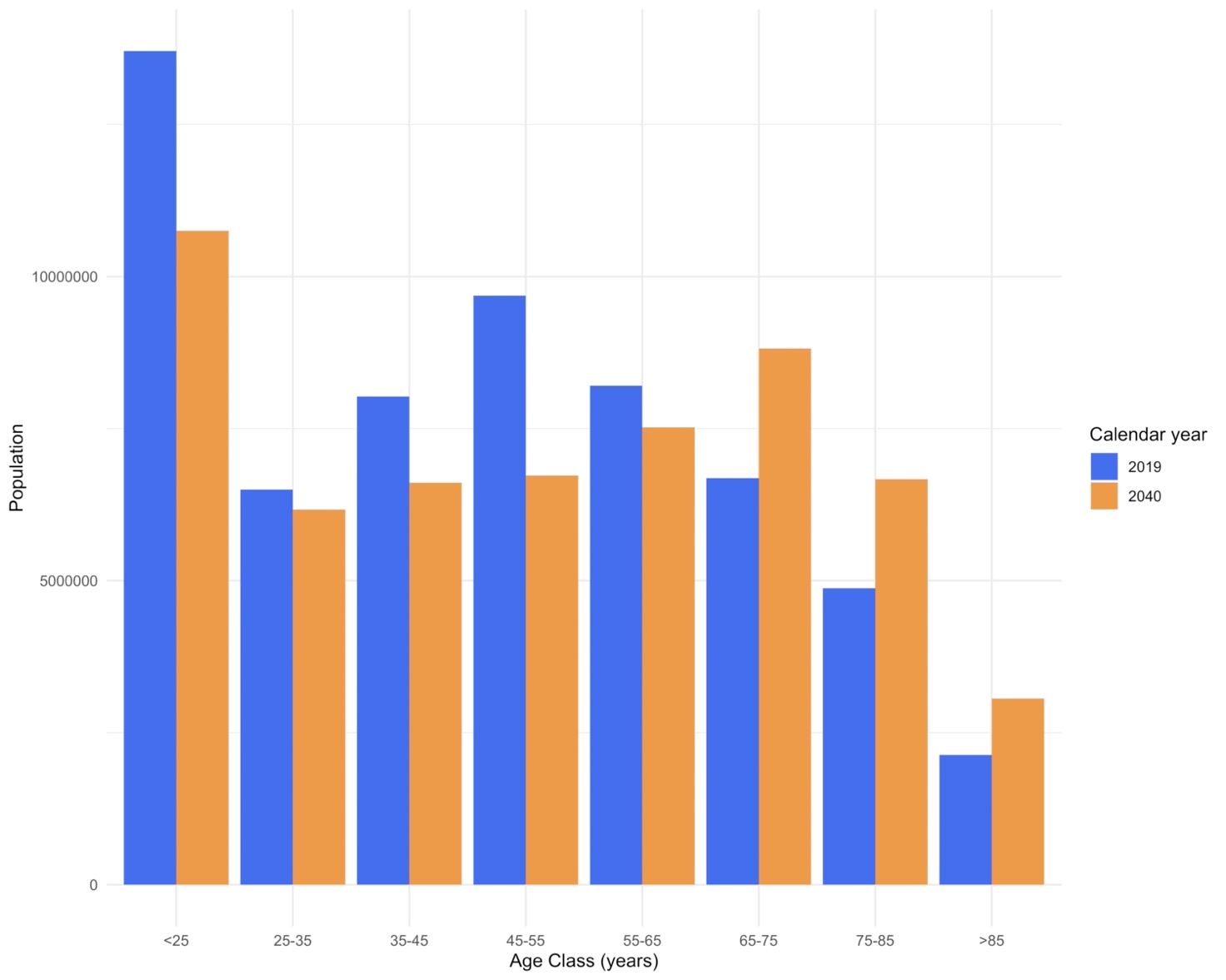


**Supplementary table 1.** Incidence, survival and prevalence projections over the PARALS area.

| **Year** | **Population** | **Incidence** | **Incident cases** | **Mean incidence** | **Survival** | **Prevalence** | **Prevalent cases** |
| --- | --- | --- | --- | --- | --- | --- | --- |
| 2005 | 4,420,042 | 3.21 | 142.0 | 3.21 | 2.47 | 7.92 | 350.1 |
| 2006 | 4,430,654 | 2.93 | 130.0 | 3.07 | 2.74 | 8.40 | 372.2 |
| 2007 | 4,483,931 | 3.35 | 150.0 | 3.16 | 2.76 | 8.74 | 391.9 |
| 2008 | 4,515,625 | 2.24 | 101.0 | 2.93 | 2.70 | 7.91 | 357.2 |
| 2009 | 4,528,529 | 3.03 | 137.0 | 2.95 | 2.78 | 8.21 | 371.8 |
| 2010 | 4,538,040 | 3.11 | 141.0 | 2.98 | 2.63 | 7.84 | 355.8 |
| 2011 | 4,544,050 | 3.30 | 150.0 | 3.02 | 2.79 | 8.45 | 384.0 |
| 2012 | 4,553,767 | 3.49 | 159.0 | 3.08 | 2.94 | 9.05 | 412.1 |
| 2013 | 4,549,169 | 2.97 | 135.0 | 3.07 | 3.01 | 9.23 | 419.9 |
| 2014 | 4,531,534 | 3,24 | 147.0 | 3.09 | 3.24 | 10.00 | 453.2 |
| 2015 | 4,511,256 | 2.82 | 127.0 | 3.06 | 3.07 | 9.40 | 424.1 |
| 2016 | 4,497,025 | 2.76 | 124.0 | 3.04 | 3.09 | 9.38 | 421.8 |
| 2017 | 4,476,124 | 3.08 | 138.0 | 3.04 | 3.27 | 9.94 | 444.9 |
| 2018 | 4,454,218 | 3.17 | 141.0 | 3.05 | 3.41 | 10.39 | 462.8 |
| 2019 | 4,436,251 | 3.61 | 160.0 | 3.09 | 3.42 | 10.55 | 468.0 |
| 2020 | 4,436,251 | 3.28 | 145.4 | 3.10 | 3.48 | 10.78 | 478.1 |
| 2021 | 4,399,034 | 3.30 | 145.3 | 3.11 | 3.54 | 11.00 | 484.1 |
| 2022 | 4,379,710 | 3.33 | 146.0 | 3.12 | 3.60 | 11.24 | 492.1 |
| 2023 | 4,374,481 | 3.35 | 146.6 | 3.14 | 3.66 | 11.47 | 501.6 |
| 2024 | 4,375,599 | 3.36 | 147.2 | 3.15 | 3.72 | 11.70 | 511.8 |
| 2025 | 4,349,439 | 3.39 | 147.3 | 3.16 | 3.78 | 11.93 | 518.9 |
| 2026 | 4,340,052 | 3.41 | 147.9 | 3.17 | 3.84 | 12.16 | 527.9 |
| 2027 | 4,329,439 | 3.44 | 149.0 | 3.18 | 3.90 | 12.40 | 536.8 |
| 2028 | 4,317,653 | 3.48 | 150.1 | 3.19 | 3.96 | 12.64 | 545.7 |
| 2029 | 4,304,661 | 3.52 | 151.4 | 3.21 | 4.02 | 12.88 | 554.5 |
| 2030 | 4,291,254 | 3.56 | 152.9 | 3.22 | 4.08 | 13.13 | 563.5 |
| 2031 | 4,278,141 | 3.61 | 154.4 | 3.23 | 4.14 | 13.38 | 572.5 |
| 2032 | 4,265,199 | 3.64 | 155.2 | 3.25 | 4.20 | 13.64 | 581.7 |
| 2033 | 4,252,377 | 3.66 | 155.8 | 3.26 | 4.26 | 13.89 | 590.8 |
| 2034 | 4,239,730 | 3.69 | 156.4 | 3.28 | 4.32 | 14.15 | 600.0 |
| 2035 | 4,227,210 | 3.72 | 157.1 | 3.29 | 4.38 | 14.41 | 609.1 |
| 2036 | 4,214,715 | 3.74 | 157.6 | 3.31 | 4.44 | 14.67 | 618.2 |
| 2037 | 4,202,269 | 3.76 | 158.2 | 3.32 | 4.50 | 14.93 | 627.4 |
| 2038 | 4,189,768 | 3.79 | 158.7 | 3.33 | 4.56 | 15.19 | 636.5 |
| 2039 | 4,177,217 | 3.81 | 159.1 | 3.35 | 4.62 | 15.45 | 645.6 |
| 2040 | 4,164,461 | 3.82 | 159.3 | 3.36 | 4.68 | 15.72 | 654.5 |

Incidence is reported as number of new cases/100,000 person-years. Survival is calculated in years. Prevalence is reported as number of cases/100,000 population. Mean incidence refers to the average of the incidence rates estimated up to a given year.

**Supplementary table 2.** Distribution of Italian population by age and sex, as it was observed in 2019 and anticipated in 2040.

|  | **2019** | |  | **2040** | |
| --- | --- | --- | --- | --- | --- |
| **Age class** | **M** | **F** |  | **M** | **F** |
| **<25** | 7,093,868 | 6,614,002 |  | 5,570,974 | 5,181,821 |
| **25-35** | 3,304,979 | 3,191,120 |  | 3,249,190 | 2,919,784 |
| **35-45** | 4,014,816 | 4,011,948 |  | 3,492,246 | 3,119,088 |
| **45-55** | 4,789,390 | 4,896,864 |  | 3,422,537 | 3,305,708 |
| **55-65** | 3,973,071 | 4,233,400 |  | 3,707,385 | 3,812,834 |
| **65-75** | 3,155,714 | 3,528,415 |  | 4,234,413 | 4,583,304 |
| **75-85** | 2,102,383 | 2,773,130 |  | 3,008,496 | 3,659,389 |
| **>85** | 696,974 | 1,436,599 |  | 1,151,933 | 1,910,002 |
| **tot** | 29,131,195 | 30,685,478 |  | 27,837,174 | 28,491,930 |

**Supplementary table 3.**  Incidence, survival and prevalence projections over the entire Italian national territory.

| **Year** | **Population** | **Incidence** | **Incident cases** | **Mean incidence** | **Survival** | **Prevalence** | **Prevalent cases** |
| --- | --- | --- | --- | --- | --- | --- | --- |
| **2019** | 59,816,673 | 3.01 | 1,799.0 | 3.01 | 3.42 | 10.28 | 6,147.5 |
| **2020** | 59,641,488 | 3.05 | 1,820.0 | 3.03 | 3.48 | 10.53 | 6,282.8 |
| **2021** | 59,236,213 | 3.09 | 1,831.1 | 3.05 | 3.54 | 10.79 | 6,390.8 |
| **2022** | 59,030,133 | 3.13 | 1,848.1 | 3.07 | 3.60 | 11.04 | 6,519.4 |
| **2023** | 58,997,201 | 3.16 | 1,865.0 | 3.09 | 3.66 | 11.29 | 6,663.7 |
| **2024** | 58,989,749 | 3.19 | 1,880.0 | 3.10 | 3-72 | 11.54 | 6,808.2 |
| **2025** | 58,732,189 | 3.22 | 1,889.5 | 3.12 | 3.78 | 11.79 | 6,923.5 |
| **2026** | 58,628,057 | 3.25 | 1,905.1 | 3.14 | 3.84 | 12.04 | 7,057.2 |
| **2027** | 58,506,609 | 3.29 | 1,925.4 | 3.15 | 3.90 | 12.29 | 7,191.7 |
| **2028** | 58,371,253 | 3.34 | 1,946.8 | 3.17 | 3.96 | 12.55 | 7,327.3 |
| **2029** | 58,229,086 | 3.38 | 1,969.2 | 3.19 | 4.02 | 12.82 | 7,464.9 |
| **2030** | 58,083,584 | 3.43 | 1,994.9 | 3.21 | 4.08 | 13.09 | 7,605.5 |
| **2031** | 57,935,995 | 3.49 | 2,019.3 | 3.23 | 4.14 | 13.37 | 7,748.3 |
| **2032** | 57,786,540 | 3.52 | 2,034.7 | 3.25 | 4.20 | 13.65 | 7,890.3 |
| **2033** | 57,634,642 | 3,55 | 2,048.5 | 3.27 | 4.26 | 13.93 | 8,031.3 |
| **2034** | 57,481,097 | 3.59 | 2,061.1 | 3.29 | 4.32 | 14.22 | 8,171.3 |
| **2035** | 57,325,883 | 3.62 | 2,074.2 | 3.31 | 4.38 | 14.50 | 8,310.5 |
| **2036** | 57,168,001 | 3.65 | 2,085.4 | 3.33 | 4.44 | 14.78 | 8,448.6 |
| **2037** | 57,007,601 | 3.68 | 2,097.3 | 3.35 | 4.50 | 15.06 | 8,585.8 |
| **2038** | 56,844,461 | 3.71 | 2,108.4 | 3.37 | 4.56 | 15.34 | 8,722.1 |
| **2039** | 56,677,541 | 3.74 | 2,117.1 | 3.38 | 4.62 | 15.63 | 8,856.9 |
| **2040** | 56,329,104 | 3.78 | 2,127.5 | 3.40 | 4.68 | 15.91 | 8,963.9 |

Incidence is reported as number of new cases/100,000 person-years. Survival is calculated in years. Prevalence is reported as number of cases/100,000 population.

Mean incidence refers to the average of the incidence rates estimated up to a given year.
